# Identifying the Four Critical Domains of the Food Protection Management System: A Pilot Study on *Ganoderma* Biotechnology Products

**DOI:** 10.1002/fsn3.72510

**Published:** 2026-10-21

**Authors:** Shuo‐Wen Tsai, Mei‐Jine Liao, Shih‐Ching Chao, Hsin‐Jung Chen

**Affiliations:** ^1^ Department of Food Science and Biotechnology National Chung Hsing University Taichung Taiwan; ^2^ Department of Food Science and Biotechnology Hung Kuang University Taichung City Taiwan; ^3^ Department of Food Science and Technology Central Taiwan University of Sciences and Technology Taichung City Taiwan

**Keywords:** food safety culture, Ganoderma biotechnology products, HACCP, risk assessment, TACCP, VACCP

## Abstract

This pilot study investigated the implementation of an integrated Food Protection Management System and evaluated food safety culture maturity within a small‐to‐medium Ganoderma biotechnology facility in Taiwan. A systematic identification methodology spanning four critical domains, HACCP, QACCP, TACCP, and VACCP, was applied across 24 processing steps. Heat sterilization and metal detection were established as Critical Control Points for unintentional hazards. Concurrently, “solid medium inoculation (mixed)” and “filling/sealing,” high‐value “strain acceptance” and “inner labeling” were identified as high‐threat and high‐vulnerability nodes for intentional threats and food fraud, leading to mitigation strategies such as molecular biological authentication and mass balance auditing. To quantitatively evaluate organizational perceptions, a food safety culture survey was conducted among 44 employees using a 20‐item scale (Cronbach's α = 0.86). The overall food safety culture maturity score reached 4.34 ± 0.65, corresponding to Level 4 (Good Awareness and Culture). An independent samples *t*‐test revealed a statistically significant perception gap (*p* < 0.05) between managerial (4.65 ± 0.48) and non‐managerial staff (4.30 ± 0.64). Furthermore, six‐month tracking revealed positive observational trends in preliminary key performance indicators, including reduced product defect and customer complaint rates. This study offers a practical reference framework for resource‐constrained biotechnology small and medium‐sized food enterprises to integrate multi‐domain protections and empirical food safety culture baselines to guide targeted employee training.

## Graphical abstract

This study investigated the implementation of a Food Protection Management System and evaluated food safety culture (FSC) maturity of a food enterprise. Four critical domains, HACCP, QACCP, TACCP, and VACCP, were applied across 24 processing steps. Heat sterilization and metal detection were established as Critical Control Points for unintentional hazards, while high‐vulnerability nodes were identified for intentional hazards. The overall FSC maturity score reached Level 4 (Good Awareness and Culture). Six‐month tracking revealed positive trends in preliminary key performance indicators.

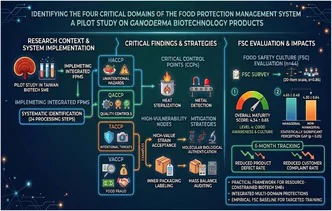

## 1 Introduction

In recent years, as global consumer health awareness has risen markedly, high‐value fungal biotechnology foods represented by *Ganoderma* (Lingzhi) have flourished in the health supplement market. Owing to their rich content of bioactive constituents such as polysaccharides and triterpenoids, these biotechnology foods are characterized by high processing technology barriers and highly attractive market economic value. However, the combination of substantial profit margins and complex multinational supply chains also renders them high‐risk targets for food fraud and intentional food defense threats (Spink and Moyer, 2011; Manning and Soon 2016). Traditionally, management systems in food plants have focused primarily on preventing unintentional microbiological, physical, or chemical contamination (i.e., the standard HACCP system). Under today's globalized and diversified threat landscape, however, a single protective system is no longer sufficient to comprehensively safeguard the authenticity, compliance, and safety of high‐value biotechnology foods.

To address the full spectrum of challenges across the food chain, international academic and industry organizations have increasingly advocated for the systematic integration of Food Safety (HACCP), Food Defense (TACCP), Food Fraud Prevention (VACCP), and quality management into a comprehensive Food Protection Management System (FPMS) (Manning and Soon 2016). Furthermore, numerous scholars have emphasized that any advanced management framework, absent an organizational food safety culture (FSC) serving as the foundation of daily behavior, risks becoming a mere formality that fails to achieve genuine implementation (Yiannas 2008; Griffith et al. 2010). Nevertheless, the existing academic literature on the integration of FPMS and FSC has predominantly emphasized macro‐level theoretical discussions or the promotion of international policy guidelines within the context of large multinational enterprises (Jespersen et al. 2017).

For small and medium‐sized food enterprises (SMFEs), which face relatively constrained resources, budgets, and technical personnel, the academic literature still lacks sufficient empirical research. Specifically, there is limited scientific evidence regarding how these diverse frameworks can be concretely localized, how consensus on hazard ratings can be achieved across multi‐stage processes, and how quantitative performance validation can be conducted following system implementation (Manning et al. 2019). This gap between academic theory and the practical implementation outcomes in SMFEs constitutes a significant research gap in the current field of biotechnology food safety management.

To address this gap, the present study adopts a representative small and medium‐sized *Ganoderma* biotechnology food manufacturer in Taiwan as the subject of a pilot study. The aim is to move beyond mere regulatory compliance descriptions or technical manuals, transforming the inquiry into a rigorous, data‐supported scientific investigation. The specific scientific objectives of this study are twofold. First, we aim to systematically establish an identification and rating methodology encompassing the four domains, including unintentional risks (HACCP, QACCP) and intentional risks (TACCP, VACCP) (Yeh et al. 2026). This approach identifies and quantifies significant hazards and vulnerabilities within the biotechnology food processing workflow. Second, through an empirical questionnaire survey, we quantitatively analyze the hierarchical differences and dynamic shifts in FSC maturity among employees. This analysis compares different departments and ranks (managerial and non‐managerial subgroups) following the system's implementation. Through this concrete pilot case analysis, this study provides the high‐value biotechnology food industry with an empirically supported systematic protection framework and academic insights applicable to SMFE management practices.

## 2 Materials and Methods

### 2.1 Study Design and Ethical Considerations

This study is a pilot observational study conducted at a small and medium‐sized *Ganoderma* biotechnology food manufacturer in Taiwan. The research structure is shown in Figure 1.

**FIGURE 1:**
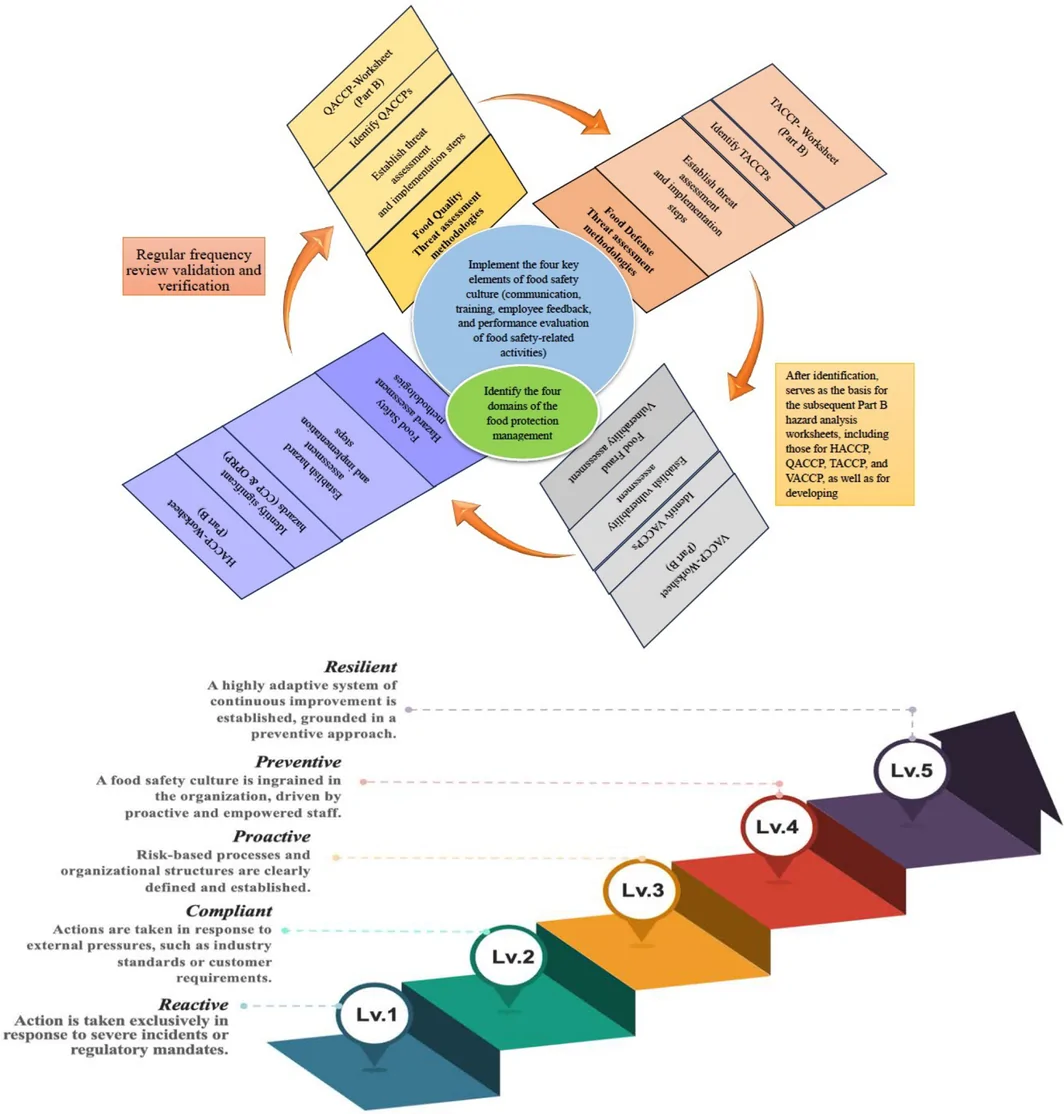
Research structure.

Regarding the employee questionnaire survey on FSC, because this study constitutes a routine internal quality management assessment and tracking of employee training effectiveness within the plant and does not involve medical procedures or invasive human experimentation, it was exempt from formal Institutional Review Board (IRB) review in accordance with standard regulations. Nonetheless, this study strictly adhered to the ethical principles for the protection of human participants set forth in the Declaration of Helsinki (World Medical Association 2013). Prior to conducting the survey, the research team fully explained the purpose of the survey to all participating employees and obtained signed informed consent from all participants. The questionnaire was administered anonymously to ensure personal privacy, and participants were explicitly informed that their responses would be used solely for academic analysis aimed at system improvement and would have no negative bearing whatsoever on their job status or performance evaluations.

### 2.2 Assessment Methodology of the Four Domains and Consensus Building

The hazard and vulnerability assessment across the four domains of the FPMS, namely HACCP, QACCP, TACCP, and VACCP, was not determined by a single external expert but was instead conducted through a comprehensive evaluation by a cross‐departmental food safety team established within the plant. This team consisted of five members, including representatives from the quality assurance (QA) department, production and manufacturing, warehousing and logistics, and senior management. During the assessment process, team members independently scored hazards and vulnerabilities using a pre‐established standardized scoring matrix (e.g., the product of severity and likelihood of occurrence), developed in accordance with the HACCP guidelines issued by the Codex Alimentarius Commission (FAO and WHO 2023) and internationally recognized standards for food defense and vulnerability assessment (e.g., PAS 96:2017) (BSI 2017). When discrepancies arose among members' scores, multiple rounds of focus group discussions were convened, in which repeated deliberation based on scientific literature, regulatory standards, and objective historical data was conducted until all team members reached consensus on the risk level for each process step.

### 2.3 Reliability Testing and Scoring Logic of the FSC Survey

To quantitatively assess employee perceptions following the implementation of the FPMS, this study drew upon an established FSC maturity model (Jespersen et al. 2017) to design a 20‐item FSC self‐assessment questionnaire. All items were scored using a 5‐point Likert scale (1 = extremely unfamiliar/dissatisfied, 5 = extremely familiar/highly satisfied). To ensure the psychometric properties of the questionnaire, the research team conducted a pre‐test prior to formal distribution and calculated Cronbach's α to confirm internal consistency reliability (Cronbach 1951).

For the maturity level classification algorithm, the total score across the 20 items completed by each employee was averaged (total score divided by 20, with a maximum of 5 points) and mapped to five maturity levels: an average score of 1.0–1.9 was classified as Level 1 (Extreme Lack of Awareness); 2.0–2.9 as Level 2 (Insufficient Awareness); 3.0–3.9 as Level 3 (Basic Awareness); 4.0–4.9 as Level 4 (Good Awareness and Culture); and 5.0 as Level 5 (Excellent Safety Culture). This quantitative conversion ensured that the classification of maturity levels was grounded in an objective mathematical basis. The content of the safety culture survey questionnaire administered to employees across all organizational levels is shown in Table 1.

**TABLE 1:** Safety culture survey questionnaire content for top‐to‐bottom department employees.

Preliminary assessment of safety culture survey			
□ Supervisor □ Non‐supervisor Department: Years of service: Gender: □ Male □ Female Date: year/month/day			
Survey items (codes 1–5 for the hurdle effect)/no		Score	Suggestions
1	Do I understand the company's food safety policies and goals?		
2	Am I confident in the company's products, and am I willing to promote them to friends and family?		
3	Do I understand the company's PRP, HACCP, VACCP, TACCP, and QACCP in my role? Am I aware of hazards and risks, and am I willing to proactively report them?		
4	Do I understand the accountability system for negligence?		
5	Do I understand the company's employee benefits or performance reward system?		
6	Do I implement tasks assigned by the leadership?		
7	Did the senior management's commitment and execution of food safety meet my expectations?		
8	Is the budget provided by senior management adequate for training, technical resources, and safety equipment?		
9	Is there an established and operational control team within the company?		
10	Does the company conduct food safety culture weeks, themed drills, or activities?		
11	Do I have access to employee health checks and a safe working environment?		
12	Is confidentiality maintained when I report food safety issues?		
13	Are the employee grievance channels, such as email or a suggestion box, effective for me?		
14	Am I satisfied with the content of my work and with my collaboration with colleagues in the same department?		
15	Am I satisfied with the content of my work and with my collaboration with colleagues in other departments?		
16	Do I understand the company's key performance indicators for annual food safety?		
17	Do I understand the company's explanation regarding the implementation of food safety culture?		
18	Do I understand the company's organizational structure and departmental responsibilities?		
19	Do I understand the company's capacity to handle change and crises, and to solve problems?		
20	Do I understand the company's PRP, HACCP, VACCP, TACCP, and QACCP in my role?		
Total score:			
Overall suggestions:	1 point: very poor understanding (or satisfaction). 2 points: poor understanding (or satisfaction). 3 points: average understanding (or satisfaction). 4 points: good understanding (or satisfaction). 5 points: excellent understanding (or satisfaction)		

### 2.4 Statistical Analysis

All questionnaire data in this study were statistically analyzed using IBM SPSS Statistics version 26.0 (IBM Corp., Armonk, NY, USA). First, descriptive statistics were used to analyze the mean scores and standard deviations (mean ± SD) of employees across departments. Second, Cronbach's α coefficient was calculated to verify the reliability of the questionnaire. Finally, to examine whether hierarchical differences existed in FSC awareness between managerial and non‐managerial employees, an independent samples *t*‐test was employed to compare group means. The significance level for all statistical tests was set at *p* < 0.05.

## 3 Results and Discussion

### 3.1 Identification of Hazards, Threats, and Vulnerabilities Across the Four Domains in the Ganoderma Processing Workflow

Through assessment by the cross‐departmental Food Safety Team, this study conducted a systematic identification of hazards (HACCP/QACCP), threats (TACCP), and vulnerabilities (VACCP) across the complete manufacturing process of the Ganoderma biotechnology product, in alignment with international food safety management guidelines (ISO 31010 2018; Global Food Safety Initiative (GFSI) 2020; FSSC 22000 2023; FAO and WHO 2023), spanning strain acceptance, liquid/solid‐state culture sterilization, drying, filling and sealing, through to inner packaging labeling. The identified significant hazards across four domains and the engineering parameter flowchart for Ganoderma biotechnology products are shown in Figure 2.

**FIGURE 2:**
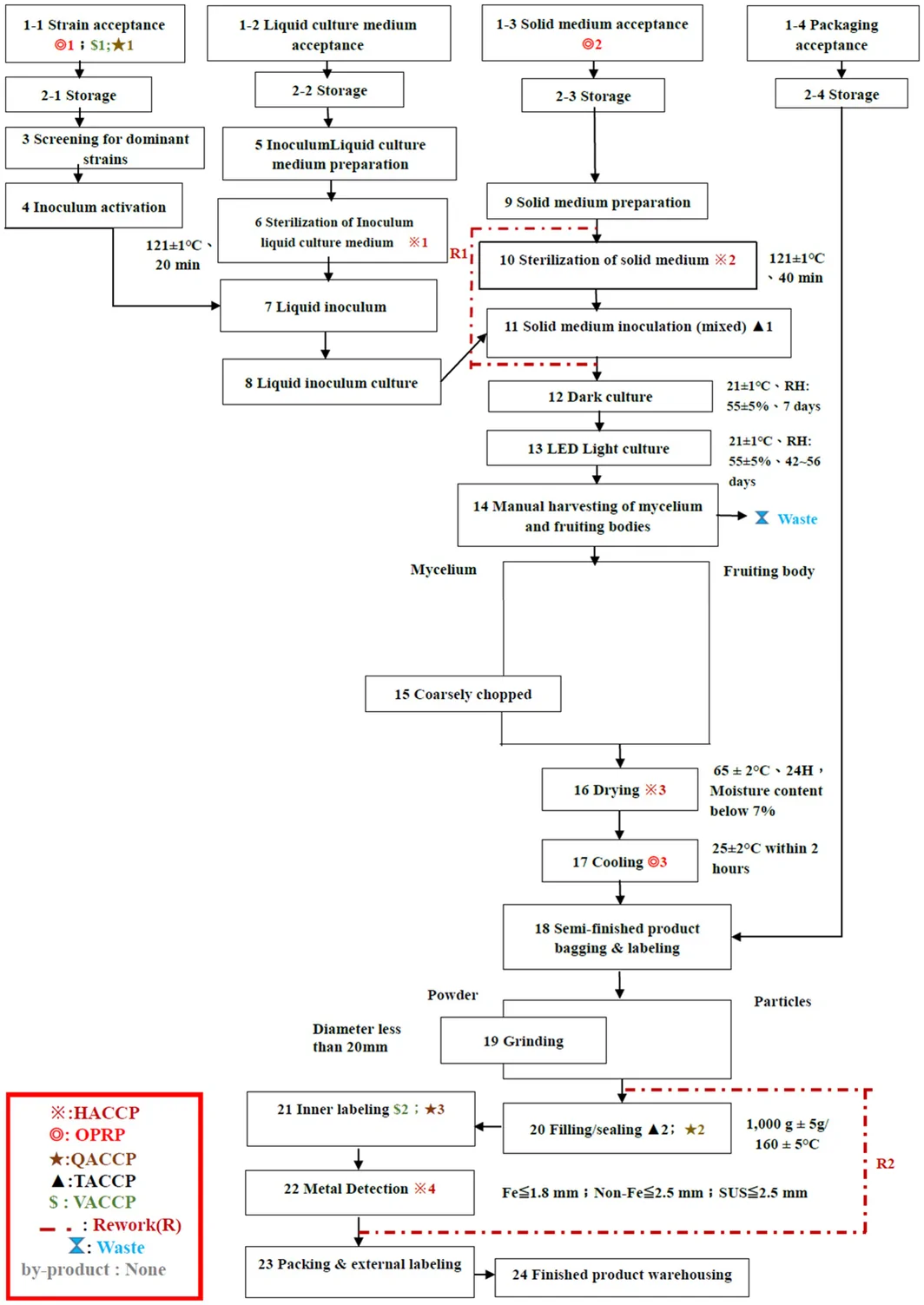
Process flow diagrams for the four domains.

With respect to unintentional food safety hazards (HACCP), the heat sterilization steps (Step 6, liquid culture medium sterilization, and Step 10, solid culture medium sterilization) were identified as Critical Control Point (CCPs), requiring heating at 121°C ± 1°C for more than 20 to 40 min to ensure effective microbial kill. The cooling (Step 17), drying (Step 16), and metal detection (Step 22) were classified as an Operational Prerequisite Program (OPRP) and a CCP, respectively, for controlling moisture content (< 7%) and preventing physical contamination from metallic foreign objects. Within the food quality (QACCP) domain, the packaging and labeling steps (Steps 20 and 21) were identified as the core focus of quality control, emphasizing the assurance of seal integrity and dosage precision (in accordance with 6σ statistical quality control rules). With regard to intentional threats (TACCP) and food fraud vulnerabilities (VACCP), this study, taking into account the specific characteristics of high‐value Ganoderma biotechnology products, identified “Solid medium inoculation(mixed) (Step 11)” and “Filling/sealing (Step 20)”, “Strain acceptance (Step 1‐1)” and “Inner labeling (Step 21)” as high‐threat and high‐vulnerability nodes separately. To prevent risks of strain adulteration, counterfeiting, or unidentified sourcing, control measures included confirming that strains were sourced from Taiwan's authorized strain collection center, conducting molecular biological authentication, establishing a supplier contract review mechanism, and implementing mass balance audits and barcode traceability systems. These identification results demonstrate that in small and medium‐sized biotechnology food plants, reliance on traditional HACCP alone is no longer sufficient to address intentional threats within modern, complex supply chains and that a complete protective barrier can only be established through the coordinated integration of the four domains. The identification of significant hazards, threats, and vulnerabilities, along with their control measures across the four critical domains, is shown in Table 2.

**TABLE 2:** Significant hazard/threat/vulnerability identification and control measures of the four critical domains.

Domain	Hazard category	Significant process step	Classification	Preliminary assessment of control measure
Food safety (FS)	Biological	Step 6: Sterilization of Inoculum Liquid Culture Medium	HACCP	Heating: 121°C ± 1°C for > 20 min
	Biological	Step 10: Sterilization of the Solid Medium		Heating: 121°C ± 1°C for > 40 min
	Biological	Step 16: Drying		Heating: 65°C ± 2°C for > 24 h, Moisture content < 7%.
	Physical	Step 22: Metal Detection		Fe 1,5 mm, non‐Fe 2,0 mm, SUS 2,5 mm check
	Biological	Step 1–1: Strain Acceptance		Verify that the strains are from an authorized strain center in Taiwan.
	Chemical	Step 1–3: Solid Medium Acceptance	OPRP	Chemical hazards and toxins meet standards, visual inspection, and supplier CoA.
	Biological	Step 17: Cooling		Cooling: 25°C ± 2°C for > 2 h
Food quality (FQ)	Packaging/Labelling	Step 1–1: Strain Acceptance		Verify that the strains are from an authorized strain center in Taiwan. Standardized scales calibration; visual check. Quality control chart (6 σ rule); visual check, seal integrity check
	Weighing	Step 20: Filling/Sealing	QACCP	
	Labelling	Step 21: Inner Labelling		Label accuracy check
Food defense (FD)	Employee's Intentional Contamination	Step 11: Solid Medium Inoculation (Mixed)	TACCP	Closed‐circuit television (CCTV) monitoring; restricted access, supervisor supervision; training & hot stove rule
	Employee's Intentional Contamination/external attack	Step 20: Filling/Sealing		Closed‐circuit television (CCTV); restricted access, supervisor supervision; training & hot stove rule
Food fraud (FF)	Substitution/ Concealment/ Counterfeiting	Step 1–1: Strain Acceptance	VACCP	Authenticity testing; supplier contract management; audit. To verify that the strains are from an authorized strain center in Taiwan. Standardized scales calibration; visual check—mass balance check; barcode verification
	Mislabeling	Step 20: Labeling		Strain identification; origin; weight; activity units; mass balance check

### 3.2 Quantitative Assessment and Hierarchical Differences in Food Safety Culture (FSC)

To verify the practical impact of FPMS implementation on employee behavior and perceptions, an FSC questionnaire survey was conducted among 44 employees, adopting a top‐down assessment approach to evaluate corporate integrity and safety awareness (Anindya and Adhariani 2019; Alrobaish et al. 2023). Reliability analysis revealed a Cronbach's α coefficient of 0.86, indicating excellent internal consistency for the self‐assessment scale. Regarding overall cultural maturity, quantitative results showed that the total average score for all employees was 4.34 ± 0.65, corresponding to Level 4 (Good Awareness and Culture) of the maturity model defined in this study.

To further explore the cognitive dynamics within the organization, an independent samples *t*‐test was employed to compare differences across management levels. The quantitative assessment of food safety culture (FSC) maturity and hierarchical differences among employees is shown in Table S1.

The results demonstrated that the average score of the managerial tier was 4.65 ± 0.48, which was significantly higher than the 4.30 ± 0.64 observed in the non‐managerial tier. This difference was statistically significant (*p* < 0.05).

These quantitative empirical results carry important management implications. The data suggest that although foundational food safety and protection awareness had been successfully established during the initial phase of FPMS implementation (with means > 4.0), a significant “perception gap” remained between the management decision‐making level and frontline operators. This finding aligns with the observations of Manning et al. (2019) regarding SMFEs: in resource‐constrained SMFEs, senior management typically possesses a more profound understanding of regulatory compliance and system architecture. However, these abstract protection concepts (e.g., VACCP and TACCP) are often difficult to rapidly and fully cascade downward to become part of the daily intuition of frontline employees. Therefore, our data strongly suggest that future system optimization and resource allocation should not be limited to establishing system documentation. Instead, highly targeted and contextualized training programs must be implemented for non‐managerial staff to bridge the hierarchical cultural gap and achieve the homogenization of protection awareness among all employees.

### 3.3 Observational Trends in Preliminary Operational Performance and Organizational Dynamics

To comprehensively evaluate the practical impacts following the implementation of FPMS and FSC, this study tracked six operational key performance indicators (KPIs), encompassing production quality, customer feedback, human resources, and cultural maturity, over a six‐month period, as shown in Figure 3A–F.

**FIGURE 3:**
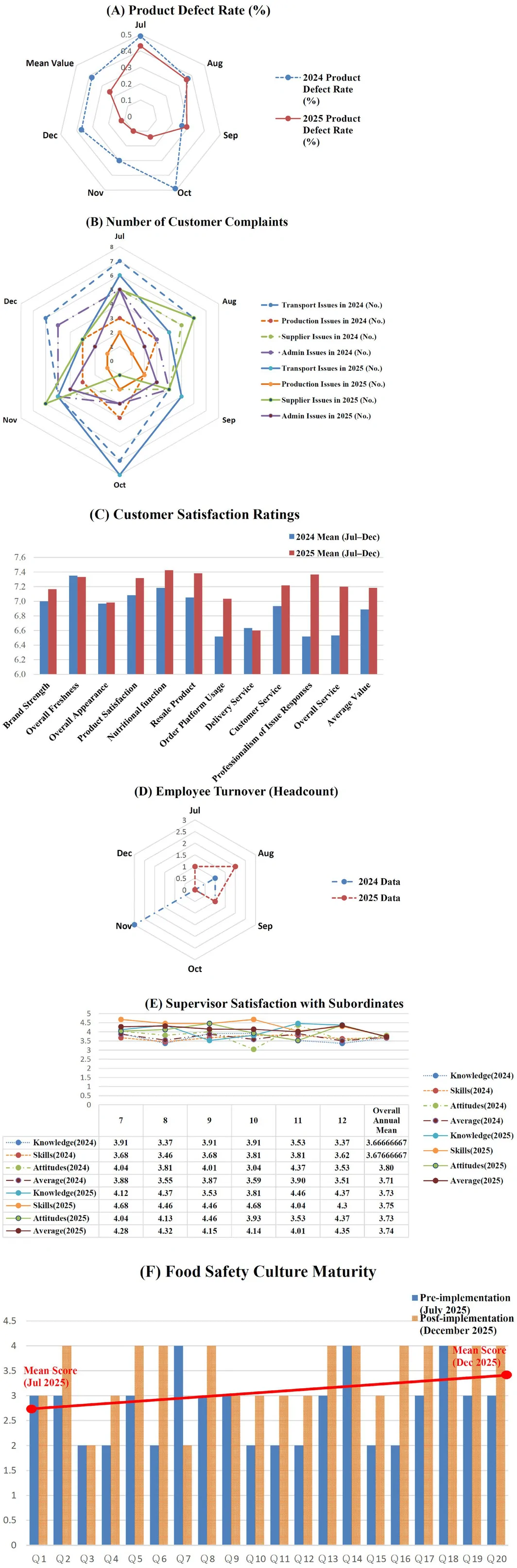
(A–F). Observational trends of Key Performance Indicators (KPIs) following the preliminary FPMS implementation.

It must be explicitly emphasized that because this is a single pilot case study lacking an independent control group, and given that operational data may be susceptible to confounding variables (e.g., seasonality or market fluctuations), the reported data should be strictly interpreted as observational trends rather than direct causal effects.

#### 3.3.1 Technical and Operational Quality Indicators

To provide an objective contextual background, the plant maintained a stable commercial production volume of approximately 3500 kg (dry basis) per month during the six‐month observation period. Under this baseline:

1. Product Defect Rate (Figure 3A): Exhibited a steady downward trend. This reduction corresponds closely with the strict implementation of CCPs and OPRPs under HACCP and QACCP (e.g., sterilization (Step 6 and 10) and drying (Step 16) (CCP), strain acceptance (Step 1–1), solid medium acceptance (Step 1–3), and cooling (Step 17) (OPRP), and seal integrity checks (Step 21)), which effectively minimized microbial and physical non‐conformities during processing.
2. Customer Complaint Rate (Figure 3B): Decreased progressively over time. This trend reflects the practical efficacy of TACCP and VACCP controls, such as packaging label audits, strain source verification, and anti‐adulteration protocols, in reducing mislabeling and product quality variations.
3. Customer Satisfaction (Figure 3C): Based on monthly feedback from a denominator of approximately 32 B2B clients (including food manufacturers and distributors), customer satisfaction demonstrated positive growth. The reduction in product variation and complaints actively fostered commercial trust among downstream B2B partners regarding raw material stability and supply chain integrity.

#### 3.3.2 Internal Organizational and Human Resource Dynamics

Regarding internal organizational management, evaluated against the baseline denominator of 44 total employees:

1. Employee Turnover Rate (Figure 3D): Showed a declining trend across the observation period. Organizational research indicates that standardized FPMS procedures and clear role definitions mitigate operational ambiguity and workplace stress among frontline workers, thereby supporting employee retention (de Andrade et al. 2020; Jespersen et al. 2019).
2. Managerial Satisfaction with Frontline Staff (Figure 3E): Supervisor satisfaction scores regarding frontline operators displayed continuous upward progress. This trend aligns with targeted training efforts; as operator compliance with CCP/OPRP monitoring improved, supervisory burden was reduced, thereby enhancing inter‐hierarchical communication and operational trust.

#### 3.3.3 Trajectory of Food Safety Culture Maturity

The overall FSC maturity score progressively advanced over the six months, stabilizing at Level 4 (Good Awareness and Culture, reaching an overall mean of 4.34 ± 0.65) (Zanin et al. 2022; PAS 320, 2023) (Figure 3F). This trajectory indicates that integrating abstract protection concepts (TACCP/VACCP) into daily HACCP practices can systematically transition an organization from passive compliance toward proactive protection awareness.

In summary, the multi‐dimensional dataset in Figure 3 highlights an interconnected positive trend: internal cultural and procedural stabilization (Figure 3D–F) translates into measurable improvements in production quality and client trust (Figure 3A–C), which supports previous findings that a mature FSC yields tangible economic and operational benefits (Jespersen et al. 2019). For resource‐constrained small and medium‐sized biotechnology food enterprises, these findings offer valuable practical insights balancing regulatory compliance with operational performance (Lee et al. 2023; Manning et al. 2024).

### 3.4 Study Limitations and Practical Implications

This study provides a practical pilot framework integrating unintentional hazards (HACCP/QACCP) and intentional threats/vulnerabilities (TACCP/VACCP), along with a preliminary quantitative evaluation of FSC maturity within a small‐to‐medium Ganoderma biotechnology plant. Nevertheless, several objective limitations must be acknowledged when interpreting these findings:

First, regarding research design and generalizability, this study was conducted as a pilot case study within a single small‐to‐medium Ganoderma biotechnology facility in Taiwan. Given that different food industry sectors (e.g., meat processing, dairy, or ready‐to‐eat foods) vary substantially in supply chain complexity, process risk profiles, and regulatory environments, the specific identification results and operational parameters of this study cannot be directly generalized across all food manufacturing domains without contextual adaptation. Second, regarding methodological limitations, operational circumstances prevented the formation of a parallel independent control group. Furthermore, the observation period for preliminary KPIs was limited to six months, and the FSC survey was restricted to the plant's total workforce of 44 employees (with *n* = 5 in the managerial subgroup), which limits broader longitudinal and high‐power causal inferences.

Regarding practical execution and resource allocation, this pilot study highlights that SMFEs frequently encounter challenges such as limited managerial bandwidth, documentation burdens, and high training costs when implementing a multi‐domain FPMS. The statistically significant perception gap observed between the managerial and non‐managerial tiers (*p* < 0.05) underscores the operational bottleneck in effectively cascading abstract defense concepts down to frontline operators. To address these limitations, this study proposes the following recommendations for future academic research and industrial practice:

- Multi‐center and Cross‐sector Validation: Future research should expand the sample size by conducting multi‐center comparative studies across SMFEs of varying operational scales and product categories to test the broader applicability of the four‐domain integration model.
- Longitudinal Assessment: Longitudinal studies spanning 12 to 24 months are recommended to track the dynamic evolution of FSC maturity and integrate quantitative risk assessment (QRA) tools to rigorously evaluate the long‐term effectiveness of system implementation.
- Contextualized Training and Simplified Assessment: Industrial managers should simplify TACCP and VACCP assessment tools and develop intuitive, highly contextualized training modules for frontline employees to effectively bridge hierarchical cognitive gaps within the organization

## 4 Conclusion

This pilot study presented an integrated FPMS framework incorporating unintentional hazards (HACCP and QACCP) and intentional threats/vulnerabilities (TACCP and VACCP) within a small‐to‐medium Ganoderma biotechnology plant in Taiwan. Across the complete manufacturing process, heat sterilization and metal detection were identified as CCPs, while strain acceptance and outer packaging labeling were designated as high‐vulnerability nodes against food fraud and intentional contamination, leading to targeted control measures.

Regarding quantitative organizational evaluation, an FSC survey among 44 employees demonstrated excellent internal consistency (Cronbach's α = 0.86), with an overall maturity score of 4.34 ± 0.65, corresponding to Level 4 (Good Awareness and Culture). Inferential statistics confirmed a statistically significant perception gap (*p* < 0.05) between managerial (4.65 ± 0.48) and non‐managerial personnel (4.30 ± 0.64), highlighting key operational bottlenecks in cascading abstract defense concepts downward to frontline staff. Additionally, preliminary six‐month KPI tracking indicated positive observational trends in reducing product defect and customer complaint rates.

In conclusion, this research provides a practical reference model combining four protection domains with quantitative cultural assessment, offering practical utility for resource‐constrained small and medium‐sized biotechnology food enterprises. Nevertheless, as a single pilot case study lacking an independent control group, the specific identification parameters and operational trends cannot be directly generalized across all food manufacturing sectors without contextual adaptation, warranting multi‐center validation and longitudinal tracking in future studies.

## Author Contributions

**Shuo‐Wen Tsai:** conceptualization, data curation, formal analysis, funding acquisition, investigation, methodology, project administration, resources, software, validation, supervision, visualization, writing – original draft, writing – review and editing. **Mei‐Jine Liao:** conceptualization, data curation, formal analysis, funding acquisition, investigation, methodology, software, validation, visualization, supervision, project administration, resources, writing – original draft. **Shih‐Ching Chao:** formal analysis, investigation, resources, validation, visualization, writing – original draft. **Hsin‐Jung Chen:** conceptualization, methodology, software, data curation, investigation, validation, formal analysis, funding acquisition, supervision, visualization, project administration, resources, writing – original draft, writing – review and editing.

## Funding

The authors have nothing to report.

## Ethics Statement

All authors have read and approved the final version of the manuscript. Hsin‐Jung Chen had full access to all of the data in this study and takes complete responsibility for the integrity of the data and the accuracy of the data analysis.

## Conflicts of Interest

The authors declare no conflicts of interest.

## Supporting information


**Table S1:** Quantitative assessment of food safety culture (FSC) maturity and hierarchical differences among employees.

## Data Availability

The data that support the findings of this study are available from the corresponding author upon reasonable request.
