# Supplementary material for: Identifying the Four Critical Domains of the Food Protection Management System: A Pilot Study on Ganoderma Biotechnology Products
Source: Food Sci Nutr. 2026 Oct 21;14(10):e72510. doi: 10.1002/fsn3.72510 (PMC13685522; doi:10.1002/fsn3.72510)
Supplement: Supplementary file 1 [file FSN3-14-e72510-s001.docx]

**Table S1.** Quantitative assessment of food safety culture (FSC) maturity and hierarchical differences among employees.

| Employee ID | Department | Gender | Role | Q1 | Q2 | Q3 | Q4 | Q5 | Q6 | Q7 | Q8 | Q9 | Q10 | Q11 | Q12 | Q13 | Q14 | Q15 | Q16 | Q17 | Q18 | Q19 | Q20 | Total Score |
| --- | --- | --- | --- | --- | --- | --- | --- | --- | --- | --- | --- | --- | --- | --- | --- | --- | --- | --- | --- | --- | --- | --- | --- | --- |
| E01 | Human Resources Department | Male | Managerial | 5 | 5 | 5 | 5 | 5 | 5 | 5 | 5 | 5 | 4 | 5 | 5 | 3 | 4 | 5 | 5 | 5 | 5 | 5 | 5 | 96 |
| E02 | Administration Department | Female | Managerial | 5 | 5 | 3 | 3 | 5 | 5 | 5 | 4 | 5 | 4 | 5 | 5 | 5 | 5 | 5 | 5 | 5 | 5 | 5 | 5 | 94 |
| E03 | Finance Department | Female | Managerial | 3 | 5 | 5 | 4 | 4 | 4 | 5 | 5 | 5 | 5 | 5 | 5 | 4 | 5 | 5 | 5 | 5 | 5 | 5 | 5 | 94 |
| E04 | Finance Department | Female | Managerial | 5 | 5 | 4 | 4 | 4 | 5 | 5 | 5 | 5 | 5 | 5 | 5 | 5 | 5 | 5 | 5 | 5 | 4 | 5 | 5 | 96 |
| E05 | Production Department | Male | Non-managerial | 5 | 4 | 4 | 4 | 4 | 4 | 5 | 5 | 4 | 4 | 3 | 4 | 3 | 5 | 3 | 4 | 4 | 4 | 4 | 5 | 82 |
| E06 | Production Department | Male | Non-managerial | 3 | 5 | 3 | 5 | 5 | 4 | 4 | 5 | 4 | 5 | 4 | 5 | 5 | 4 | 5 | 5 | 5 | 4 | 4 | 5 | 89 |
| E07 | Production Department | Male | Non-managerial | 4 | 5 | 4 | 3 | 5 | 4 | 4 | 5 | 4 | 5 | 4 | 5 | 5 | 5 | 5 | 5 | 5 | 5 | 5 | 5 | 92 |
| E08 | Production Department | Male | Non-managerial | 5 | 5 | 5 | 4 | 4 | 5 | 4 | 5 | 4 | 5 | 4 | 5 | 5 | 5 | 5 | 4 | 4 | 4 | 4 | 4 | 90 |
| E09 | Production Department | Male | Non-managerial | 5 | 5 | 5 | 5 | 5 | 4 | 4 | 5 | 5 | 3 | 4 | 5 | 4 | 5 | 5 | 5 | 4 | 4 | 5 | 5 | 92 |
| E10 | Production Department | Male | Non-managerial | 5 | 5 | 5 | 4 | 4 | 5 | 5 | 5 | 5 | 5 | 5 | 5 | 3 | 5 | 4 | 5 | 4 | 4 | 4 | 5 | 92 |
| E11 | Production Department | Male | Non-managerial | 5 | 5 | 5 | 5 | 3 | 5 | 5 | 5 | 4 | 4 | 4 | 5 | 4 | 5 | 5 | 4 | 4 | 4 | 3 | 4 | 88 |
| E12 | Production Department | Male | Non-managerial | 5 | 5 | 5 | 4 | 5 | 5 | 5 | 5 | 5 | 5 | 4 | 5 | 5 | 5 | 5 | 5 | 5 | 4 | 5 | 4 | 96 |
| E13 | Production Department | Male | Non-managerial | 4 | 5 | 4 | 4 | 4 | 5 | 5 | 4 | 4 | 4 | 4 | 5 | 4 | 5 | 4 | 4 | 5 | 4 | 4 | 4 | 86 |
| E14 | Production Department | Male | Non-managerial | 4 | 5 | 4 | 4 | 4 | 5 | 5 | 5 | 5 | 4 | 5 | 5 | 5 | 4 | 4 | 4 | 4 | 5 | 4 | 4 | 89 |
| E15 | Production Department | Male | Non-managerial | 4 | 5 | 4 | 4 | 4 | 4 | 4 | 5 | 4 | 4 | 5 | 5 | 5 | 4 | 4 | 4 | 4 | 4 | 4 | 4 | 85 |
| E16 | Packaging Team | Female | Non-managerial | 4 | 5 | 5 | 4 | 4 | 4 | 4 | 4 | 3 | 4 | 4 | 4 | 4 | 4 | 4 | 5 | 4 | 4 | 4 | 4 | 82 |
| E17 | Packaging Team | Male | Non-managerial | 4 | 4 | 4 | 3 | 3 | 4 | 5 | 4 | 4 | 4 | 3 | 4 | 5 | 4 | 4 | 4 | 3 | 4 | 4 | 4 | 78 |
| E18 | Packaging Team | Female | Non-managerial | 4 | 4 | 4 | 3 | 3 | 3 | 3 | 5 | 3 | 3 | 4 | 5 | 3 | 3 | 3 | 3 | 3 | 3 | 3 | 4 | 69 |
| E19 | Packaging Team | Female | Non-managerial | 5 | 4 | 4 | 4 | 3 | 4 | 3 | 4 | 3 | 4 | 4 | 5 | 3 | 3 | 3 | 4 | 4 | 4 | 3 | 4 | 75 |
| E20 | Packaging Team | Female | Non-managerial | 5 | 5 | 5 | 5 | 4 | 5 | 5 | 5 | 5 | 3 | 5 | 5 | 5 | 5 | 4 | 4 | 5 | 5 | 5 | 5 | 95 |
| E21 | Packaging Team | Female | Non-managerial | 5 | 5 | 5 | 3 | 3 | 5 | 5 | 4 | 5 | 4 | 5 | 5 | 3 | 4 | 4 | 5 | 4 | 4 | 4 | 4 | 86 |
| E22 | Quality Control Department | Female | Non-managerial | 4 | 5 | 4 | 4 | 3 | 4 | 4 | 4 | 4 | 4 | 5 | 5 | 5 | 4 | 4 | 5 | 4 | 4 | 4 | 5 | 85 |
| E23 | Quality Control Department | Female | Non-managerial | 4 | 5 | 4 | 4 | 3 | 3 | 4 | 4 | 3 | 4 | 4 | 5 | 3 | 3 | 4 | 5 | 4 | 4 | 3 | 5 | 78 |
| E24 | Quality Control Department | Female | Non-managerial | 5 | 5 | 5 | 5 | 5 | 5 | 4 | 5 | 5 | 4 | 5 | 5 | 3 | 4 | 4 | 5 | 4 | 4 | 4 | 4 | 90 |
| E25 | R&D Team | Male | Managerial | 4 | 5 | 4 | 4 | 4 | 4 | 4 | 4 | 3 | 5 | 5 | 5 | 5 | 3 | 4 | 5 | 3 | 4 | 5 | 5 | 85 |
| E26 | R&D Team | Male | Non-managerial | 5 | 5 | 4 | 4 | 3 | 5 | 4 | 3 | 3 | 3 | 4 | 5 | 5 | 3 | 3 | 3 | 4 | 4 | 4 | 4 | 78 |
| E27 | Warehousing Team | Female | Non-managerial | 4 | 5 | 5 | 4 | 3 | 5 | 4 | 3 | 3 | 3 | 4 | 4 | 3 | 3 | 3 | 3 | 3 | 3 | 4 | 4 | 73 |
| E28 | Logistics Team | Male | Non-managerial | 4 | 4 | 3 | 4 | 5 | 4 | 5 | 4 | 3 | 3 | 5 | 5 | 5 | 4 | 4 | 5 | 4 | 4 | 3 | 5 | 83 |
| E29 | Logistics Team | Male | Non-managerial | 4 | 4 | 5 | 4 | 5 | 4 | 4 | 3 | 5 | 3 | 5 | 5 | 5 | 3 | 5 | 5 | 4 | 4 | 4 | 5 | 86 |
| E30 | Logistics Team | Male | Non-managerial | 4 | 5 | 3 | 4 | 5 | 5 | 4 | 4 | 4 | 4 | 4 | 5 | 3 | 3 | 3 | 4 | 4 | 4 | 3 | 5 | 80 |
| E31 | Logistics Team | Male | Non-managerial | 5 | 5 | 5 | 4 | 4 | 5 | 4 | 5 | 5 | 4 | 5 | 4 | 5 | 5 | 4 | 5 | 5 | 5 | 4 | 5 | 93 |
| E32 | Sales Department | Male | Non-managerial | 4 | 5 | 4 | 4 | 5 | 5 | 4 | 5 | 3 | 4 | 4 | 4 | 5 | 5 | 4 | 5 | 3 | 4 | 4 | 4 | 85 |
| E33 | Sales Department | Female | Non-managerial | 4 | 5 | 4 | 4 | 5 | 5 | 4 | 4 | 4 | 4 | 4 | 5 | 4 | 3 | 4 | 4 | 4 | 4 | 4 | 4 | 83 |
| E34 | Marketing Team | Male | Non-managerial | 5 | 5 | 5 | 4 | 4 | 5 | 4 | 4 | 4 | 4 | 5 | 4 | 5 | 4 | 4 | 5 | 4 | 4 | 4 | 5 | 88 |
| E35 | Marketing Team | Female | Non-managerial | 4 | 5 | 5 | 5 | 5 | 4 | 4 | 5 | 4 | 3 | 5 | 5 | 5 | 5 | 5 | 5 | 5 | 4 | 5 | 5 | 93 |
| E36 | Business Operations Department | Female | Non-managerial | 4 | 4 | 4 | 3 | 3 | 4 | 4 | 4 | 4 | 4 | 4 | 4 | 3 | 3 | 3 | 4 | 4 | 4 | 4 | 4 | 75 |
| E37 | Business Operations Department | Female | Non-managerial | 5 | 5 | 4 | 4 | 3 | 5 | 4 | 3 | 3 | 3 | 4 | 5 | 5 | 3 | 3 | 3 | 4 | 4 | 4 | 4 | 78 |
| E38 | Business Operations Department | Female | Non-managerial | 5 | 5 | 4 | 4 | 4 | 5 | 4 | 4 | 5 | 5 | 4 | 5 | 4 | 5 | 4 | 5 | 4 | 4 | 5 | 5 | 90 |
| E39 | Ingredients Preparation Team | Male | Non-managerial | 3 | 5 | 5 | 5 | 5 | 4 | 5 | 5 | 3 | 3 | 5 | 5 | 5 | 5 | 5 | 5 | 5 | 5 | 5 | 5 | 93 |
| E40 | Ingredients Preparation Team | Female | Non-managerial | 3 | 5 | 5 | 4 | 5 | 5 | 5 | 5 | 5 | 5 | 5 | 5 | 5 | 5 | 5 | 5 | 5 | 4 | 5 | 4 | 95 |
| E41 | Ingredients Preparation Team | Female | Non-managerial | 3 | 5 | 4 | 5 | 5 | 4 | 5 | 5 | 3 | 5 | 5 | 5 | 5 | 5 | 3 | 3 | 4 | 4 | 5 | 5 | 88 |
| E42 | Engineering & Maintenance Team | Male | Non-managerial | 5 | 5 | 4 | 5 | 5 | 4 | 4 | 4 | 5 | 5 | 4 | 5 | 4 | 5 | 4 | 5 | 4 | 4 | 5 | 5 | 91 |
| E43 | Engineering & Maintenance Team | Male | Non-managerial | 5 | 5 | 4 | 4 | 5 | 5 | 5 | 5 | 4 | 3 | 5 | 5 | 5 | 3 | 4 | 4 | 4 | 4 | 5 | 5 | 89 |
| E44 | Filling Preparation Team | Male | Non-managerial | 3 | 5 | 5 | 4 | 5 | 5 | 5 | 5 | 5 | 5 | 5 | 5 | 5 | 5 | 5 | 5 | 5 | 4 | 4 | 5 | 95 |
| Average | | | Overall (*n*=44) | 4.32 ± 0.71 | 4.84 ± 0.37 | 4.34 ± 0.64 | 4.09 ± 0.60 | 4.18 ± 0.81 | 4.50 ± 0.59 | 4.39 ± 0.58 | 4.45 ± 0.66 | 4.11 ± 0.81 | 4.05 ± 0.75 | 4.45 ± 0.59 | 4.82 ± 0.39 | 4.32 ± 0.86 | 4.20 ± 0.85 | 4.14 ± 0.73 | 4.48 ± 0.70 | 4.20 ± 0.63 | 4.14 ± 0.46 | 4.23 ± 0.68 | 4.57 ± 0.5 | 4.34 ± 0.65 |
|  |  |  | Managerial (*n*=5) | 4.40 ± 0.89 | 5.00 ± 0.00 | 4.20 ± 0.84 | 4.00 ± 0.71 | 4.40 ± 0.55 | 4.60 ± 0.55 | 4.80 ± 0.45 | 4.60 ± 0.55 | 4.60 ± 0.89 | 4.60 ± 0.55 | 5.00 ± 0.00 | 5.00 ± 0.00 | 4.40 ± 0.89 | 4.40 ± 0.89 | 4.80 ± 0.45 | 5.00 ± 0.00 | 4.60 ± 0.89 | 4.60 ± 0.55 | 5.00 ± 0.00 | 5.00 ± 0.00 | 4.65 ± 0.48 |
|  |  |  | Non-managerial (*n*=39) | 4.31 ± 0.69 | 4.82 ± 0.39 | 4.36 ± 0.63 | 4.10 ± 0.6 | 4.15 ± 0.84 | 4.49 ± 0.60 | 4.33 ± 0.58 | 4.44 ± 0.68 | 4.05 ± 0.79 | 3.97 ± 0.74 | 4.38 ± 0.59 | 4.79 ± 0.41 | 4.31 ± 0.86 | 4.18 ± 0.85 | 4.05 ± 0.72 | 4.41 ± 0.72 | 4.15 ± 0.59 | 4.08 ± 0.42 | 4.13 ± 0.66 | 4.51 ± 0.51 | 4.30 ± 0.64 |
| *p*-value | | | | > 0.05 | < 0.01** | > 0.05 | > 0.05 | > 0.05 | > 0.05 | > 0.05 | > 0.05 | > 0.05 | > 0.05 | < 0.001*** | < 0.01** | > 0.05 | > 0.05 | < 0.05* | < 0.001*** | > 0.05 | > 0.05 | < 0.001*** | < 0.001*** | < 0.05* |

Note: Data are expressed as mean ± standard deviation (SD) (*n* = 44). The maturity scores were evaluated using a 20-item questionnaire based on a 5-point Likert scale (1 = extremely unfamiliar/dissatisfied, 5 = extremely familiar/highly satisfied). Differences between the managerial and non-managerial subgroups were analyzed using an independent samples t-test. The overall internal consistency reliability of the questionnaire was confirmed with a Cronbach's α of 0.86. *p* < 0.05 indicates statistical significance.
